# The Association of Antibiotic Stewardship With Fluoroquinolone Prescribing in Michigan Hospitals: A Multi-hospital Cohort Study

**DOI:** 10.1093/cid/ciy1102

**Published:** 2019-02-13

**Authors:** Valerie M Vaughn, Tejal Gandhi, Anna Conlon, Vineet Chopra, Anurag N Malani, Scott A Flanders

**Affiliations:** 1 Division of Hospital Medicine, Department of Internal Medicine, Michigan Medicine, Ann Arbor, Michigan; 2 Center for Clinical Management Research, Veterans Affairs Ann Arbor Health System, Ann Arbor, Michigan; 3 Division of Infectious Diseases, Department of Internal Medicine, Michigan Medicine, Ann Arbor, Michigan; 4 Division of Infectious Diseases, Department of Internal Medicine, St. Joseph Mercy Health System, Ann Arbor, Michigan; 5 Department of Infection Prevention and Control, St. Joseph Mercy Health System, Ann Arbor, Michigan

**Keywords:** antibiotic stewardship, fluoroquinolone, transitions of care, pneumonia, urinary tract infection

## Abstract

**Background:**

Fluoroquinolones increase the risk of *Clostridioides difficile* infection and antibiotic resistance. Hospitals often use pre-prescription approval or prospective audit and feedback to target fluoroquinolone prescribing. Whether these strategies impact aggregate fluoroquinolone use is unknown.

**Methods:**

This study is a 48-hospital, retrospective cohort of general-care, medical patients hospitalized with pneumonia or positive urine culture between December 2015–September 2017. Hospitals were surveyed on their use of pre-prescription approval and/or prospective audit and feedback to target fluoroquinolone prescribing during hospitalization (fluoroquinolone stewardship). After controlling for hospital clustering and patient factors, aggregate (inpatient and post-discharge) fluoroquinolone (ciprofloxacin, levofloxacin, moxifloxacin) exposure was compared between hospitals with and without fluoroquinolone stewardship.

**Results:**

There were 11 748 patients (6820 pneumonia; 4928 positive urine culture) included at 48 hospitals. All hospitals responded to the survey: 29.2% (14/48) reported using pre-prescription approval and/or prospective audit and feedback to target fluoroquinolone prescribing. After adjustment, fluoroquinolone stewardship was associated with fewer patients receiving a fluoroquinolone (37.1% vs 48.2%; *P* = .01) and fewer fluoroquinolone treatment days per 1000 patients (2282 vs 3096 days/1000 patients; *P* = .01), driven by lower inpatient prescribing. However, most (66.6%) fluoroquinolone treatment days occurred after discharge, and hospitals with fluoroquinolone stewardship had twice as many new fluoroquinolone starts after discharge as hospitals without (15.6% vs 8.4%; *P* = .003).

**Conclusions:**

Hospital-based stewardship interventions targeting fluoroquinolone prescribing were associated with less fluoroquinolone prescribing during hospitalization, but not at discharge. To limit aggregate fluoroquinolone exposure, stewardship programs should target both inpatient and discharge prescribing.

## Teaser

In this 48-hospital cohort study of 11 748 patients hospitalized with pneumonia or positive urine cultures, hospital-based antibiotic stewardship interventions targeting fluoroquinolone prescribing were associated with less fluoroquinolone use during hospitalization, but appeared to partially increase fluoroquinolone prescribing at discharge.

Up to half of hospitalized patients receive antibiotics, most often for pneumonia or a urinary tract infection (UTI) [1]. Of these, 1 in 5 will develop an antibiotic-associated adverse event [2]. In particular, fluoroquinolone antibiotics (ciprofloxacin, levofloxacin, moxifloxacin) are associated with severe adverse reactions and the development of antibiotic resistance [3, 4]. Regional rates of fluoroquinolone prescribing (defined daily doses) are highly correlated with regional rates of *Clostridioides difficile* infection (CDI) [5]. For example, the United Kingdom’s decrease in *C. difficile* was likely driven by reduced fluoroquinolone prescribing [5]. Although up to half of patients hospitalized with pneumonia or a UTI receive a fluoroquinolone, safer alternatives often exist [6].

To mitigate the risks related to fluoroquinolone use, antibiotic stewardship guidelines recommend that hospitals reduce fluoroquinolone prescribing [4]. Specifically, 2 core stewardship interventions are strongly recommended: pre-prescription approval (ie, requiring approval prior to prescribing restricted antibiotics) and/or prospective audit and feedback (ie, providing feedback to providers after prescribing) [4, 7, 8]. Use of these core, hospital-based interventions to target fluoroquinolone prescribing in hospitals (ie, fluoroquinolone stewardship) has been shown to reduce inpatient fluoroquinolone prescribing [7–11]. However, their effect on aggregate (inpatient and post-discharge) fluoroquinolone prescribing is unknown. Therefore, we evaluated the association of fluoroquinolone stewardship with aggregate fluoroquinolone use in patients hospitalized with pneumonia or a positive urine culture in a 48-hospital cohort study.

## METHODS

### Study Setting and Participants

This retrospective cohort study included 48 diverse hospitals that were participating in the Michigan Hospital Medicine Safety (HMS) Consortium (a collaborative quality initiative) between December 2015 and September 2017. Participation in HMS is voluntary, and includes more than half (52%, 48/92) of the hospitals in Michigan, including rural hospitals, small (<200 beds) and large (>200 beds) community hospitals, and academic teaching hospitals.

Patients eligible for inclusion were adult, non–intensive care, medical patients hospitalized with pneumonia or a positive urine culture. Patients who were pregnant, severely immunocompromised, or had concomitant infections were excluded.

Pneumonia was identified by International Classification of Diseases (ICD) discharge diagnostic codes, and confirmed by: positive radiographic findings, ≥2 symptoms of pneumonia, and receipt of antibiotic treatment by hospital day 2 (to increase the specificity of ICD codes) [12]. Patients were included if they had community-acquired pneumonia (CAP) or health-care–associated pneumonia (HCAP; at least 1 of the following: nursing home resident, hospitalization within 90 days, intravenous chemotherapy, home wound care or home infusion therapy, or chronic hemodialysis within 30 days) [13]. Patients with hospital-acquired or ventilator-associated pneumonia were excluded. Patients with a positive urine culture included both patients with symptoms attributable to a UTI and those without symptoms (asymptomatic bacteriuria). Positive urine cultures included any urine culture flagged as positive by that hospital. Urine cultures positive only for a *Candida* species (without a concurrent bacterial pathogen) were not included; otherwise, cultures were not excluded based on colony-forming units or the number/type of pathogen. Patients with urological procedures during the hospitalization were excluded (Supplementary Table 1).

Daily discharge lists at each hospital were consecutively sampled and screened by trained abstractors, who included 2 patients (1 with pneumonia and 1 with a positive urine culture) daily at each hospital [14].

### Data Collection

Detailed patient data, including inpatient antibiotic administration and discharge prescriptions, were collected from the medical record by trained abstractors from 90 days prior to admission until follow-up was terminated by a major complication (transfer to intensive care, death) or until 30 days after discharge.

Additionally, hospitals were surveyed annually on program characteristics and stewardship interventions. HMS-supported abstractors (who work at each hospital) were asked to identify the best person/people (eg, stewardship or quality lead) to respond to each survey question and ensure data accuracy. Hospitals are incentivized to accurately complete surveys as part of a pay-for-performance program funded by Blue Cross and Blue Shield of Michigan. Specifically, hospitals were asked the following questions:

1. Does a physician or pharmacist review targeted antimicrobials (ie, prospective audit with feedback) at your facility? (If yes, which ones?)
2. Do specific antibiotic agents need to be approved by a physician or a pharmacist prior to dispensing (ie, pre-authorization) at your facility? (If yes, which ones?)

### Data Analysis

The primary outcomes of interest were: (1) the proportion of patients prescribed a fluoroquinolone (as an inpatient or after discharge) and (2) the aggregate number of days of fluoroquinolone therapy per 1000 patients (as inpatients and after discharge). Patients were determined to have received a fluoroquinolone after discharge if there was an electronic, paper, or phone outpatient prescription for a fluoroquinolone at the time of hospital discharge. If no outpatient prescription could be identified, the treatment plan documented in the discharge summary was reviewed to determine antibiotic treatment after discharge. Fluoroquinolone antibiotics included ciprofloxacin, levofloxacin, and moxifloxacin: the main fluoroquinolones used in the United States. Hospitals were considered to use fluoroquinolone stewardship if they: (1) reported use of pre-prescription approval and/or prospective audit and feedback and (2) included 1 or more fluoroquinolones (or all antibiotics) in their list of targeted agents. Hospitals with fluoroquinolone stewardship were compared to hospitals without. For patients who received fluoroquinolones, the secondary outcome was the duration of fluoroquinolone therapy. Sensitivity analyses examined the effect of location (eg, inpatient only, started as an inpatient and continued after discharge, or after discharge only) and disease state (eg, pneumonia, positive urine culture) on the primary outcomes.

The proportions of patients receiving a fluoroquinolone were compared using generalized estimating equation models, taking into account hospital clustering. The aggregate days of fluoroquinolone therapy per 1000 patients and the durations of fluoroquinolone therapy were compared using negative binomial generalized estimating equation models. Patients who did not have an antibiotic duration documented at discharge were excluded from the analyses of aggregate days and duration of therapy. All analyses controlled for patient characteristics that could potentially affect the rates of fluoroquinolone prescribing, including age, gender, sepsis on admission, Charlson comorbidity index, Medicaid insurance, race, allergy to penicillin or cephalosporins, length of stay, diagnosis (CAP, HCAP, UTI, pyelonephritis, asymptomatic bacteriuria), and bacteremia. There were minimal missing data; for the 2 variables with some missing data (insurance: 5.8%, 676/11 748; race: 1.4%, 166/11 748 patients), values were imputed through a 10-fold multiple imputation procedure and combined using standard rules for multiple imputation [15]. We also conducted a sensitivity analysis without imputing missing data [15]. We set a *P* value <.05 as significant. SAS version 9.4 was used for analyses.

### Ethics Statement

As the purpose of the HMS consortium is to measure and improve the quality of existing care practices, it received a “not regulated” status by the University of Michigan institutional review board.

## RESULTS

A total of 11 748 patients in 48 hospitals (6820 with pneumonia [4463 with CAP; 2357 with HCAP] and 4928 with a positive urine culture) were eligible and included in this analysis. All hospitals responded to the survey and completed questions related to pre-prescription approval and/or prospective audit and feedback (response rate 100%). Nearly all hospitals (46/48, 96%) reported having an antibiotic stewardship program. While most hospitals performed pre-prescription approval (77%, 37/48) and/or prospective audit and feedback (90%, 43/48), only 29% (14/48) reported fluoroquinolone(s) among their list of targeted antibiotics (1 hospital: pre-prescription approval; 10 hospitals: prospective audit and feedback; 3 hospitals: both). Patients at hospitals with fluoroquinolone stewardship were younger (70.5 vs 74.3 years old, *P* = .03), more likely to have Medicaid (11.5% vs 7.1%, *P* = .03), and more likely to have pyelonephritis (Table 1).

**Table 1.** Characteristics of Hospitals With and Without Fluoroquinolone Stewardship, Bivariable Analysis, N = 48 Hospitals

	Hospitals Without Fluoroquinolone- directed Stewardship (n = 34)	Hospitals With Fluoroquinolone- directed Stewardship (n = 14)	*P* Value
**Hospital characteristics, n (%)**			
Academic^a^	15 (44%)	8 (57%)	.41
Location			.23
Metropolitan	24 (71%)	13 (93%)	
Micropolitan	7 (21%)	1 (7%)	
Rural	3 (9%)	0 (0%)	
Profit type			.07
Non-profit	29 (85%)	8 (57%)	
For profit	3 (9%)	2 (14%)	
Governmental	2 (6%)	4 (29%)	
Bed size			.82
Median (IQR)	305 (186–391)	278 (136–584)	
Mean (SD)	328 (242)	365 (264)	
<50 Beds	0 (0)	0 (0)	
51–100 Beds	4 (12%)	2 (14%)	
101–200 Beds	6 (18%)	4 (29%)	
>200 Beds	24 (71%)	8 (57%)	
Have an antimicrobial stewardship program	33 (97%)	13 (93%)	.51
**Case mix,^b^ median (IQR)**			
Age	74.3 (72.4–75.7)	70.5 (66.7–74.8)	.03
Percentage of patients who were female	60.0 (56.5–63.3)	60.2 (56.2–61.3)	.52
Percentage of patients with sepsis on admission	49.4 (40.2–54.1)	53.3 (45.0–56.9)	.27
Percentage of patients with Charlson Comorbidity Index ≥ 3	56.6 (50.4–61.5)	57.4 (54.3–60.9)	.61
Percentage of patients with Medicaid insurance	7.1 (4.9–9.9)	11.5 (5.5–16.6)	.03
Percentage of non-white patients	9.5 (1.4–17.4)	15.7 (4.2–76.3)	.18
Percentage of cases with documented allergy to penicillin or cephalosporin	9.7 (7.4–12.0)	8.4 (5.6–10.6)	.22
Length of stay	5 (4–5)	4.5 (4–5)	.49
Percentage of patients with bacteremia^c^	2.7 (1.5–3.5)	3.4 (2.4–4.6)	.08
Percentage of patients with CAP	36.7 (32.6–43.3)	35.8 (34.0–40.2)	.71
Percentage of patients with HCAP	18.2 (15.9–22.4)	19.9 (16.4–25.7)	.42
Percentage of patients with urinary tract infection	28.2 (21.0–32.9)	31.7 (25.2–37.0)	.18
Percentage of patients with asymptomatic bacteriuria	13.8 (9.3–19.7)	12.0 (8.8–13.6)	.16
Percentage of patients with pyelonephritis^d^	3.1 (0.7–4.4)	4.1 (3.5–6.9)	.01

The data presented are from a comparison of hospital demographics (obtained by chart review) and stewardship factors (obtained by annual surveys) in hospitals with and without fluoroquinolone stewardship (pre-prescription approval or prospective audit and feedback).

Abbreviations: CAP, community-acquired pneumonia; HCAP, health-care–associated pneumonia; IQR, inter-quartile range; SD, standard deviation.

^a^Self-reported.

^b^Includes N = 11 748 patients at 48 hospitals.

^c^Does not include blood cultures growing only organisms frequently associated with contamination.

^d^Pyelonephritis was diagnosed by a positive urine culture plus documentation of flank pain or costovertebral angle tenderness.

An antibiotic was prescribed at discharge for 71.5% (8394/11 748) of patients. Fluoroquinolone antibiotics were the most common antibiotic class prescribed at discharge. In all, 30.5% (3582/11 748) of patients were prescribed a fluoroquinolone at discharge, including 28.1% (3304/11 748) who had an outpatient prescription for a fluoroquinolone identified at the time of discharge; 0.2% (25/11 748) who had no prescription, but had planned discharge fluoroquinolone treatment documented in their discharge summary; and 2.2% (253/11 748) who had a fluoroquinolone name, but not a duration, listed at the time of discharge and, thus, were excluded from analyses of aggregate days and durations of therapy (Supplementary Figure 1).

Across hospitals, 42.6% (5001/11 748) of patients were prescribed a fluoroquinolone during hospitalization or after discharge, including 46.8% (2090/4463) of patients with CAP, 52.7% (1242/2357) with HCAP, 36.5% (1238/3396) with a UTI, and 28.1% (431/1532) with asymptomatic bacteriuria. For patients with CAP or HCAP, levofloxacin was the most commonly prescribed fluoroquinolone (86.6%, 2884/3332). For patients with a positive urine culture, ciprofloxacin was the most common (68.2%, 1139/1669).

### Location of Fluoroquinolone Prescribing

**Figure 1.**
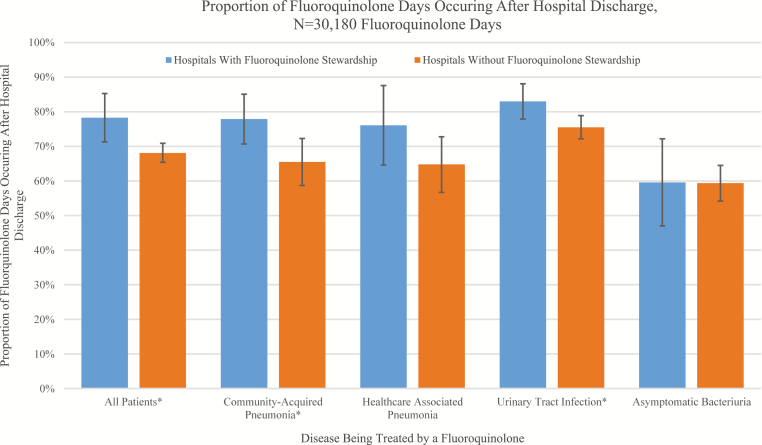
Proportion of fluoroquinolone treatment days occurring after hospital discharge, N = 30 180 fluoroquinolone treatment days

Two-thirds (66.6%, 20 112/30 180) of total fluoroquinolone treatment days occurred after discharge. After adjusting for hospital clustering and patient characteristics, a greater proportion of fluoroquinolone treatment days occurred after discharge in hospitals with fluoroquinolone stewardship (78.3% [5803/7411], 95% confidence interval [CI] 71.3–85.3%) than in hospitals without (68.1% [15 506/22 769], 95% CI 65.4–70.9%; *P* = .02; Figure 1). While few patients (18.8% [654/3479], 95% CI 13.2–24.4%) at hospitals without fluoroquinolone stewardship were first started on a fluoroquinolone after discharge, nearly half (45.8% [581/1268], 95% CI 33.7–58.0%) of patients who received a fluoroquinolone in hospitals with fluoroquinolone stewardship were started after discharge.

### Primary Outcome

Following adjustment, fewer patients received a fluoroquinolone (inpatient or after discharge) at hospitals with fluoroquinolone stewardship than at hospitals without (37.1% [1302/3510], 95% CI 30.3–43.9%, vs 48.2% [3971/8238], 95% CI 44.4–51.9%, respectively; *P* = .01). In addition, there were fewer total days of fluoroquinolone therapy per 1000 patients at hospitals with vs without fluoroquinolone stewardship (2282 days/1000 patients, 95% CI 1799–2765 days/1000 patients, vs 3096 days/1000 patients, 95% CI 2818–3374/1000 patients, respectively; *P* = .01; Table 2).

**Table 2.** Fluoroquinolone Exposure in Hospitalized Patients With Pneumonia or a Positive Urine Culture

	Proportion Receiving Fluoroquinolone, % (95% CI), [n/N] (n = 11 748)			Fluoroquinolone Treatment Days per 1000 Patients^a^ (95% CI) (n = 11 495)		
	Without Fluoroquinolone Stewardship: 8238 Patients, 34 Hospitals	With Fluoroquinolone Stewardship: 3510 Patients, 14 Hospitals	*P* Value	Without Fluoroquinolone Stewardship: 8023 Patients, 34 Hospitals	With Fluoroquinolone Stewardship: 3472 Patients, 14 Hospitals	*P* value
Any location (inpatient or after discharge)	48.2% (44.4–51.9%) [3971/8238]	37.1% (30.3–43.9%) [1302/3510]	.01	3096 (2818–3374)	2282 (1799–2765)	.01
Inpatient only	15.5% (11.4–19.6%) [1277/8238]	8.8% (4.1–13.5%) [309/3510]	.06	317 (258–376)	163 (83–242)	.01
Started as inpatient and continued after discharge	22.3% (19.8–24.8%) [1837/8238]	10.3% (7.4–13.1%) [362/3510]	<.001	1895 (1675–2115)	977 (654–301)	<.001
After discharge only	8.4% (6.4–10.4%) [692/8238]	15.6% (10.9–20.4%) [548/3510]	.003	627 (507–748)	1033 (743–1324)	.005

All data were adjusted for clustering by hospital and patient characteristics. Fluoroquinolone stewardship includes pre-prescription approval and/or prospective audit and feedback targeting fluoroquinolone prescribing in the hospital.

Abbreviation: CI, confidence interval.

^a^Excludes patients lacking a documented discharge duration of fluoroquinolone therapy (253/11 748, 2.2%).

After adjustment, hospitals with fluoroquinolone stewardship generally had less inpatient fluoroquinolone use (Table 2). However, there was no difference after discharge in the proportion of patients receiving fluoroquinolones (28.0%, 95% CI 21.6–34.4% vs 31.8%, 95% CI 29.0–34.6%, respectively; *P* = .36) or fluoroquinolone treatment days per 1000 patients (1734 days/1000 patients, 95% CI 1319–2149/1000 patients, vs 1970 days/1000 patients, 95% CI 1767–2174 days/1000 patients, respectively; *P* = .35) for hospitals with vs without fluoroquinolone stewardship. Additionally, hospitals with fluoroquinolone stewardship had twice as many new starts of fluoroquinolone therapy after discharge as hospitals without fluoroquinolone stewardship (15.6% [548/3510], 95% CI 10.9–20.4%, vs 8.4% [692/8238], 95% CI 6.4–10.4%, respectively; *P* = .003) and more discharge-only fluoroquinolone treatment days per 1000 patients (Table 2).

### Secondary Outcomes

Adjusted total durations of fluoroquinolone treatment for those who started as an inpatient and continued after discharge or those who started after discharge only were not significantly different between patients who received fluoroquinolones at hospitals with vs hospitals without fluoroquinolone stewardship. While hospitals with fluoroquinolone stewardship had statistically significant shorter inpatient-only fluoroquinolone durations, the absolute difference was less than 0.1 days (Supplementary Table 2).

### Association of Fluoroquinolone Stewardship With Fluoroquinolone Prescribing by Disease State

The results were similar to the primary analysis when patients with pneumonia were analyzed separately: fewer total patients received a fluoroquinolone and there were fewer total fluoroquinolone treatment days per 1000 patients in hospitals with vs without fluoroquinolone stewardship (Table 3). However, when patients with a positive urine culture were analyzed separately, hospitals with fluoroquinolone stewardship had fewer total patients receive a fluoroquinolone, but no significant difference in the total fluoroquinolone treatment days per 1000 patients (2902/1000 patients, 95% CI 2594–3211/1000 patients, vs 2252/1000 patients, 95% CI 1718–2786/1000 patients, respectively; *P* = .07), compared to hospitals without fluoroquinolone stewardship. Hospitals with fluoroquinolone stewardship had double the number of new fluoroquinolone starts after discharge in patients hospitalized with pneumonia, compared to hospitals without fluoroquinolone stewardship (16.8% [357/2126], 95% CI 10.2–23.4%, vs 7.8% [366/4694], 95% CI 5.2–10.3%, respectively; *P* = .001; Table 3).

**Table 3.** Fluoroquinolone Exposure in Hospitalized Patients: Subgroup Analysis by Disease State

	Pneumonia (n = 6820)						Positive Urine Culture (n = 4928)					
	Proportion Receiving Fluoroquinolone % (95% CI) [n/N]			Fluoroquinolone Treatment Days per 1000 Patients^a^ (95% CI)			Proportion Receiving Fluoroquinolone % (95% CI) [n/N]			Fluoroquinolone Treatment Days per 1000 Patients^a^ (95% CI)		
	Without Fluoroquinolone Stewardship: 4694 Patients, 34 Hospitals	With Fluoroquinolone Stewardship: 2126 Patients, 14 Hospitals	*P* Value	Without Fluoroquinolone Stewardship: 4559 Patients, 34 Hospitals	With Fluoroquinolone Stewardship: 2094 Patients, 14 Hospitals	*P* Value	Without Fluoroquinolone Stewardship: 3544 Patients, 34 Hospitals	With Fluoroquinolone Stewardship: 1384 Patients, 14 Hospitals	*P* Value	Without Fluoroquinolone Stewardship: 3464 Patients, 34 Hospitals	With Fluoroquinolone Stewardship: 1378 Patients, 14 Hospitals	*P* Value
Any location (inpatient or after discharge)	55.2% (49.1–61.3%) [2591/4694]	43.1% (34.9–51.4%) [916/2126]	.01	3540 (3038–4043)	2456 (1899–3013)	.004	45.2% (41.4–49.0%) [1602/3544]	34.3% (28.0–40.5%) [475/1384]	.005	2902 (2594–3211)	2252 (1718–2786)	0.07
Inpatient only	21.1% (14.0–28.3%) [990/4694]	9.1% (3.9–14.2%) [193/2126]	.01	426 (309–543)	181 (85–277)	.01	10.8% (8.4–13.2%) [383/3544]	8.5% (4.8–12.1%) [118/1384]	.31	246 (188–304)	158 (84–231)	0.07
Started as inpatient and continued after discharge	28.0% (23.4–32.6%) [1314/4694]	13.2% (9.2–17.1%) [281/2126]	<.001	2363 (1909–2816)	1084 (791–1376)	<.001	20.1% (17.4–22.7%) [712/3544]	9.7% (6.1–13.2%) [134/1384]	<.001	1674 (1430–1919)	941 (529–1353)	0.02
After discharge only	7.8% (5.2–10.3%) [366/4694]	(16.8% (10.2–23.4%) [357/2126]	.001	532 (376–687)	1027 (639–1415)	<.001	9.7% (7.8–11.7%) [344/3544]	12.8% (9.9–15.8%) [177/1384]	.04	691 (537–845)	904 (590–1218)	0.19

All data were adjusted for clustering by hospital and patient characteristics. Fluoroquinolone stewardship includes pre-prescription approval and/or prospective audit and feedback targeting fluoroquinolones.

Abbreviation: CI, confidence interval.

^a^Excludes patients lacking a documented discharge duration of fluoroquinolone therapy (253/11 748, 2.2%).

Overall, the findings were similar (eg, total proportion of patients receiving a fluoroquinolone; fluoroquinolone treatment days per 1000 patients) in a sensitivity analysis where missing values were not imputed, with the exception that the proportion of patients receiving an inpatient-only fluoroquinolone was significantly lower in hospitals with fluoroquinolone stewardship (8.3% [291/3510], 95% CI 4.8–14.0%, vs 16.0% [1318/8238], 95% CI 11.6–21.5%, respectively; *P* = .03; Supplementary Table 3).

## DISCUSSION

In this multicenter, observational study of 11 748 hospitalized patients with pneumonia or a positive urine culture, fluoroquinolone use was common, especially after discharge. Hospital-based fluoroquinolone stewardship was associated with less inpatient, but not post-discharge, fluoroquinolone use. Although fewer patients received fluoroquinolones in hospitals with fluoroquinolone stewardship, high levels of new fluoroquinolone starts after discharge attenuated the effect of fluoroquinolone stewardship on the total days of fluoroquinolone therapy. Thus, fluoroquinolone stewardship appeared to partially shift fluoroquinolone prescribing from the inpatient to post-discharge setting.

Consistent with prior studies and reviews, we found that targeting fluoroquinolone prescribing using a core stewardship intervention (pre-prescription approval and/or prospective audit and feedback) was associated with less inpatient fluoroquinolone use [7–9, 11, 16]. However, we found these interventions had an attenuated association with aggregate fluoroquinolone exposure, which is linked to population rates of CDI and resistance [5, 17]. Specifically, fluoroquinolone stewardship was not associated with less fluoroquinolone exposure in patients with a positive urine culture, even with adjustment for patient characteristics that could be associated with appropriate fluoroquinolone use (eg, bacteremia, pyelonephritis). This is especially concerning, as nearly a third of patients in this group had asymptomatic bacteriuria, which does not require antibiotic treatment and is challenging for inpatient stewardship teams to identify, and yet many patients were treated with fluoroquinolones [18]. Furthermore, in their “Boxed Warnings,” the US Food and Drug Administration now recommends against using fluoroquinolones for uncomplicated UTIs, unless there are no alternative treatment options [19]. Although fluoroquinolone stewardship was associated with less fluoroquinolone prescribing in the subgroup of patients hospitalized with pneumonia, fluoroquinolone use for these patients remained high, and new fluoroquinolone starts after discharge were more common.

Despite alternative therapies during hospitalization, we found that many patients were switched to a fluoroquinolone at discharge. This could be due to the innate advantages of fluoroquinolones after discharge (eg, single agent, oral bioavailability, daily dosing, low cost), but is also potentially due to limited provider knowledge regarding harms and alternatives. Some switching at discharge may be appropriate: for example, as providers change to oral therapy when treating resistant organisms. However, providers may also be switching to fluoroquinolone antibiotics at discharge to avoid stewardship policies they perceive as overly restrictive, a practice known as “stealth dosing” [20]. As fluoroquinolone use for even 1–3 days can double the risk of CDI [3], this switch at discharge could have negative consequences for patients.

Prior studies have suggested that antibiotic prescribing at discharge may account for half of all antibiotic use related to acute hospitalizations for infections and may account for most excess durations [21, 22]. We found that discharge prescribing appears critical for fluoroquinolones, with up to 80% of fluoroquinolone treatment days occurring after discharge in hospitals with fluoroquinolone stewardship. Thus, antibiotic stewardship programs that do not track discharge prescribing may not adequately capture aggregate fluoroquinolone exposure. In the United States currently, standardized hospital antibiotic use data reported to the National Healthcare Safety Network do not capture antibiotics prescribed on discharge [23, 24]. Appropriate antibiotic prescribing at discharge may become an even more critical target in the future, as lengths of stay decrease and more antibiotic treatment occurs after discharge [25].

Our study has limitations. First, this is observational, making it susceptible to confounding. While we adjusted for many factors associated with fluoroquinolone use, including antibiotic allergies, we could not adjust for all factors, such as antibiotic resistance. Second, although a third of inpatient fluoroquinolone use is typically inappropriate [6], we did not assess for the appropriateness of fluoroquinolone prescribing or for compensatory increases in other potentially inappropriate antibiotic classes. Rather, we focused on the aggregate benefits of reducing fluoroquinolone use [5]. Notably, any antibiotic use for asymptomatic bacteriuria is inappropriate and, thus, fluoroquinolone use for this group is unambiguously inappropriate. Future studies should evaluate the effect of core stewardship strategies on appropriate vs inappropriate fluoroquinolone prescribing and the compensatory increases in other antibiotic classes. Third, we focused on the 2 most effective, common, and guideline-recommended core interventions to reduce fluoroquinolone prescribing [4, 26], but hospitals may have been using other interventions (eg, education, disease-based interventions) to reduce fluoroquinolone prescribing. Fourth, we did not evaluate the strength of strategy implementation and, due to a low number of hospitals using pre-prescription approval alone, were unable to compare strategies. Although robust pre-prescription approval or prospective audit and feedback can be more efficacious, especially if targeting discharge [22, 26], our study provides a real-world snapshot of these interventions as currently practiced by stewardship programs. Fifth, this is a single-state study, which may limit generalizability. Sixth, we relied on self-reported practices by hospitals. Although we incentivized hospitals to provide survey responses and share responses with the collaborative, which may increase the likelihood of accurate reporting, we were unable to confirm actual stewardship practices. Finally, we could not assess patient compliance with prescriptions and, thus, may overestimate fluoroquinolone exposure. Study strengths include manual data abstraction by trained abstractors, which enhanced the reliability of the data related to antibiotic prescriptions after discharge; use of a large number of hospitals; and inclusion of the 2 most common indications for fluoroquinolone prescribing (pneumonia and positive urine culture). To our knowledge, this is the first study to evaluate inpatient plus discharge fluoroquinolone prescribing across multiple hospitals.

Our study has important implications. Given the frequency of fluoroquinolone prescribing after discharge, hospital-based stewardship programs should implement discharge stewardship, or interventions to address discharge antibiotic prescribing. To do so, interventions must combat the advantages of fluoroquinolones, perhaps by focusing on their disadvantages (eg, side effects, resistance, contraindications) and offering alternatives. To start, institutional treatment guidelines should: (1) recommend specific, alternative, narrow-spectrum antibiotics after discharge, when appropriate, (2) educate providers on these guidelines, and (3) build recommendations into order sets and the reporting of results, to nudge providers away from fluoroquinolone use [27]. Discharge stewardship should be paired with other effective stewardship interventions. For example, audit and feedback could intentionally focus on transitions of care, to reduce fluoroquinolone prescribing [22]. In addition, broad interventions to reduce excessive antibiotic duration may also improve fluoroquinolone prescribing at discharge, especially in cases where antibiotics may be stopped prior to leaving the hospital. Furthermore, pairing fluoroquinolone stewardship with disease-based (eg, asymptomatic bacteriuria) interventions may reduce fluoroquinolone use further, by eliminating unnecessary antibiotic use generally. Finally, given the magnitude and importance of aggregate fluoroquinolone exposure on the rates of CDI [5], national measures of antibiotic use should consider including antibiotic prescribing both during hospitalization and at discharge.

In summary, hospital-based fluoroquinolone stewardship was associated with less inpatient fluoroquinolone use, but appeared to partially shift fluoroquinolone prescribing to discharge, attenuating its association with aggregate fluoroquinolone exposure. By failing to address antibiotic prescribing at discharge, stewardship interventions may limit their impact on patient safety.

## Supplementary Data

Supplementary materials are available at *Clinical Infectious Diseases* online. Consisting of data provided by the authors to benefit the reader, the posted materials are not copyedited and are the sole responsibility of the authors, so questions or comments should be addressed to the corresponding author.

ciy1102_suppl_Supplementary_Tables_1-3

ciy1102_suppl_Supplementary_Figure_1

## Notes

***Acknowledgments.* **The authors thank Elizabeth McLaughlin for editorial support, in addition to her work as project manager for the Michigan Hospital Medicine Safety Consortium, and David Ratz for support with data verification and analysis.

***Disclaimer.* **Blue Cross and Blue Shield of Michigan supported data collection at each participating site and funded the data coordinating center, but had no role in the study design; collection, analysis, and interpretation of data; writing of the report; or decision to submit the paper for publication. V. M. V. had full access to all the data in the study and final responsibility for the decision to submit for publication.

***Financial support.* **This work was supported by Blue Cross and Blue Shield of Michigan and Blue Care Network, as part of their Value Partnerships program.

***Potential conflicts of interest.* **All authors: No reported conflicts. All authors have submitted the ICMJE Form for Disclosure of Potential Conflicts of Interest. Conflicts that the editors consider relevant to the content of the manuscript have been disclosed.
