# Supplementary material for: The Association of Antibiotic Stewardship With Fluoroquinolone Prescribing in Michigan Hospitals: A Multi-hospital Cohort Study
Source: Clin Infect Dis. 2019 Feb 13;69(8):1269–77. doi: 10.1093/cid/ciy1102 (PMC6763628; doi:10.1093/cid/ciy1102)
Supplement: ciy1102_suppl_Supplementary_Tables_1-3 [file ciy1102_suppl_supplementary_tables_1-3.docx]

| eTable 1. Sampling, Inclusion, and Exclusion Criteria | | |
| --- | --- | --- |
| Cohort | Patients with Pneumonia | Patients with a Positive Urine Culture |
| Inclusion Criteria | Hospitalized, adult (≥18 years), general care, medical patients without a concomitant infection | |
| Sampling Frame | Patients with ICD discharge diagnosis of pneumonia, positive radiographic findings, and antibiotic administration by hospital day 2 (increases specificity of ICD code and excludes hospital-acquired pneumonia) | All positive urine cultures in hospitalized patients |
| Sampling Method | At each hospital, daily discharge list was consecutively sampled until 1 patient daily met criteria for enrollment per condition | |
| Exclusions | Admitted to intensive care unit, pregnant, severe immune compromise (e.g., acquired immunodeficiency syndrome, transplant), concomitant infection, transferred from another hospital, previously included admission in prior 30 days, comfort care/hospice, left against medical advice | |
| Disease-specific Exclusions | Ventilator-associated or hospital-acquired pneumonia, cystic fibrosis, fungal pneumonia, mycobacterial infections | Urologic procedure, stent, or nephrostomy tube during hospitalization or prior urinary diversion surgery (treatment recommendations vary), on suppressive antibiotic therapy for chronic urinary tract infections |
| Variables Collected from the Medical Record | Patient variables: demographics, admission symptoms, physical exam findings, daily vital signs, microbiology results, laboratory (e.g., white blood cell count, procalcitonin) and radiology results, comorbidities, immune suppressing medications, procedures, payer  Antibiotic data: inpatient antibiotic administration, discharge prescriptions (directly via orders or from treatment plan documented in discharge summary) | |
| Variables Collected by Annual Stewardship Survey | Stewardship variables: current antibiotic stewardship activities, guidelines, and policies  Organization variables: academic, bed size, profit, urbanicity | |

Abbreviations. ICD, International Classification of Diseases

**eTable 2.** Duration of Fluoroquinolone Therapy, by location, in Hospitalized Patients with Pneumonia or a Positive Urine Culture (N=4,747)

| **Setting of Fluoroquinolone Use** | **Duration of Fluoroquinolone Therapy; Median (IQR), Days** | | |
| --- | --- | --- | --- |
|  | **Without Fluoroquinolone Stewardship**  **(3479 patients, 34 hospitals)** | **With Fluoroquinolone Stewardship**  **(1268 patients, 14 hospitals)** | **P Value** |
| Any Location (Inpatient or After Discharge) | 6.4 (3.6, 8.8) | 5.3 (3.5, 7.4) | 0.21 |
| Inpatient Only | 2.5 (1.0, 4.1) | 2.5 (1.0, 4.0) | 0.04 |
| Started Inpatient AND Continued After Discharge | 8.5 (6.9, 10.4) | 7.1 (5.4, 9.5) | 0.23 |
| After Discharge Only | 5.4 (5.0, 7.4) | 5.5 (4.1, 7.5) | 0.10 |

All data adjusted for clustering by hospital and patient characteristics. Includes only patients who received a fluoroquinolone. Fluoroquinolone stewardship includes pre-prescription approval and/or prospective audit and feedback targeting fluoroquinolone prescribing in the hospital. Patients with an antibiotic name, but missing a discharge duration, were excluded from analyses (N=253/11,748 [2.2%]).

**eTable 3.** Fluoroquinolone Exposure in Hospitalized Patients with Pneumonia or a Positive Urine Culture, Sensitivity Analysis without Multiple Imputation

|  | **Proportion Receiving Fluoroquinolone; % (95% CI), [N/N]**  **N=10,933** | | | **Fluoroquinolone Days per 1000 Patients^a^ (95% CI)**  **N=10,699** | | |
| --- | --- | --- | --- | --- | --- | --- |
|  | **Without Fluoroquinolone Stewardship**  **(7529 patients, 33 hospitals)** | **With Fluoroquinolone Stewardship**  **(3404 patients, 14 hospitals)** | **P Value** | **Without Fluoroquinolone Stewardship**  **(7333 patients, 33 hospitals)** | **With Fluoroquinolone Stewardship**  **(3366 patients, 14 hospitals)** | **P Value** |
| Any Location (Inpatient or Discharge) | 48.3% (44.5%, 51.2%)  [3979/8238] | 36.7% (30.2%, 43.7%)  [1288/3510] | 0.008 | 3103 (2828, 3405) | 2242 (1819, 2762) | 0.007 |
| Inpatient Only | 16.0% (11.6%, 21.5%)  [1318/8238] | 8.3% (4.8%, 14.0%)  [291/3510] | 0.03* | 320 (265, 388) | 162 (99, 265) | 0.01 |
| Started Inpatient AND Continued After Discharge | 22.5% (20.1%, 25.2%)  [1854/8238] | 10.1% (7.6%, 13.3%)  [355/3510] | <0.001 | 1922 (1709, 2161) | 955 (689, 1324) | <0.001 |
| Discharge Only | 8.2% (6.3%, 10.6%)  [676/8238] | 15.2% (11.1%, 20.6%)  [534/3510] | 0.004 | 611 (503, 741) | 996 (755, 1313) | 0.006 |

All data adjusted for clustering by hospital and patient characteristics when missing data are not imputed.

* Indicates difference in statistical significance compared to main analysis. Fluoroquinolone stewardship includes pre-prescription approval and/or prospective audit and feedback targeting fluoroquinolone prescribing in the hospital.

^a^ Excludes patients lacking a documented discharge duration of fluoroquinolone therapy (N=253/11,748 [2.2%]).

Abbreviations. CI, confidence interval
