# Supplementary figures and images for: The Association of Antibiotic Stewardship With Fluoroquinolone Prescribing in Michigan Hospitals: A Multi-hospital Cohort Study
Source: Clin Infect Dis. 2019 Feb 13;69(8):1269–77. doi: 10.1093/cid/ciy1102 (PMC6763628; doi:10.1093/cid/ciy1102)

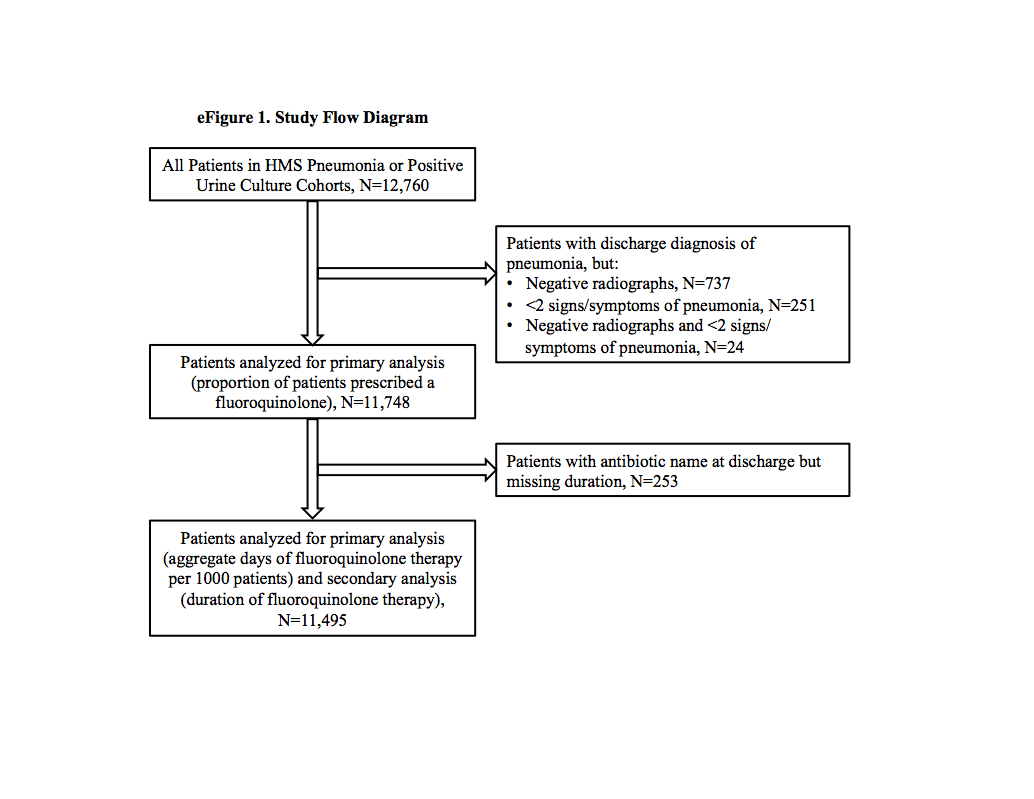

Supplement: ciy1102_suppl_Supplementary_Figure_1 [file ciy1102_suppl_supplementary_figure_1.png]
